# Remaining Useful Life Prediction Method for Stochastic Degrading Devices Considering Predictive Maintenance

**DOI:** 10.3390/s25041218

**Published:** 2025-02-17

**Authors:** Qing Dong, Hong Pei, Changhua Hu, Jianfei Zheng, Dangbo Du

**Affiliations:** The Department of Automation, Rocket Force University of Engineering, Xi’an 710025, China; 18756528162@163.com (Q.D.); hch66603@163.com (C.H.); zjf302@126.com (J.Z.); ddb_effort@126.com (D.D.)

**Keywords:** predictive maintenance, remaining useful life, repairable degrading devices, imperfect maintenance, adaptive Wiener, sensor-based monitoring

## Abstract

Predictive maintenance, recognized as an effective health management strategy for extending the lifetime of devices, has emerged as a hot research topic in recent years. A general method is to execute two separate steps: data-driven remaining useful life (RUL) prediction and a maintenance strategy. However, among the numerous studies that conducted maintenance and replacement activities based on the results of RUL prediction, little attention has been paid to the impact of preventive maintenance on sensor-based monitoring data, which further affects the RUL for repairable degrading devices. In this paper, an adaptive RUL prediction method is proposed for repairable degrading devices in order to improve the accuracy of prediction results and achieve adaptability to future degradation processes. Firstly, a phased degradation model based on an adaptive Wiener process is established, taking into account the impact of imperfect maintenance. Meanwhile, integrating the impact of maintenance activities on the degradation rate and state, the probability distribution of RUL can be derived based on the concept of first hitting time (FHT). Secondly, a method is proposed for model parameter identification and updating that incorporates the individual variation among devices, integrating maximum likelihood estimation and Bayesian inference. Finally, the effectiveness of the RUL prediction method is ultimately validated through numerical simulation and its application to repairable gyroscope degradation data.

## 1. Introduction

The emergence of Industry 4.0 has facilitated the advancement of sophisticated devices, such as aviation industry machinery, missile weapon systems, and high-speed trains, toward intelligence and integration. Consequently, the timely and effective maintenance of devices is imperative to ensure their safe and reliable operation [1,2]. Prognostic and health management technology, as a health management activity involving monitoring, prediction, and maintenance, has garnered substantial attention from both academia and industry in recent years [3,4,5]. In comparison to traditional failure maintenance and planned maintenance approaches, predictive maintenance (PdM) leverages forecasted information of remaining useful life (RUL) to implement maintenance strategies, thereby reducing operation and maintenance costs while ensuring the safe and reliable operation of devices [6].

The data-driven method serves as the fundamental and pivotal component for PdM decision-making, encompassing both statistical data-driven methods and machine learning-based methods [7,8]. By leveraging extensive monitoring data pertaining to the health status of systems, the machine learning-based method can obtain the predictive information of devices’ RUL, thereby formulating more efficient maintenance strategies [9]. For instance, Nguyen et al. [10] initially presented a dynamic PdM framework that utilizes deep learning for failure prognostics. In this framework, a long short-term memory network is employed to estimate the probability of system failure, enabling real-time decision-making by promptly evaluating maintenance and spare parts costs. To elucidate prognostic uncertainties, Zhuang et al. [11] proposed a Bayesian deep learning-based prognostic driven dynamic PdM framework that incorporates the latest predictive RUL information to update maintenance and spare part ordering decisions. This method exhibits the advantages of excellent universality and low implementation costs, but it is difficult to quantify the inherent uncertainty associated with the degradation process.

The statistical data-driven method aims to utilize statistical processes, such as the Wiener process, Gamma process, and inverse Gaussian process, to construct a degradation model that describes the trajectory of devices’ degradation [10,12,13]. In contrast to the machine learning-based method, it can derive the probability density function (PDF) of RUL and facilitate subsequent maintenance activities. Specifically, due to its mathematical properties and nonmonotonicity, the degradation model based on the Wiener process has attracted widespread attention from academia and industry [14,15,16]. However, in the aforementioned PdM studies, it is commonly assumed that prediction and maintenance are relatively independent components, i.e., that the prediction results only serve as guidance for maintenance activities, while neglecting the influence of maintenance activities on RUL prediction throughout the entire lifecycle. If preventive maintenance is carried out before the device’s failure, it can effectively reduce the operational risk of devices and extend their RUL. Therefore, it is imperative to study the RUL prediction problem while incorporating maintenance activities.

Based on the description of maintenance activities in previous work [17], they can typically be categorized as perfect maintenance, minor repair, and imperfect maintenance. Specifically, perfect maintenance reinstates degraded indicators that reflect the device’s health condition to the pristine state, which is difficult to achieve in practical engineering. Minor repairs can only slightly improve the health condition of devices to a certain extent. Imperfect maintenance can partially mitigate the device’s degraded condition but falls short of fully restoring it to an optimal state, occupying a position between minor repairs and perfect maintenance. Compared to perfect maintenance and minor repairs, the majority of preventive maintenance in practical engineering is classified as imperfect maintenance based on industry standards, which has attracted extensive attention from researchers. For example, Kijima [18] and Nakagawa [19] utilized the virtual age model and the risk increase model, respectively, to depict the impact of imperfect maintenance on degradation processes. Based on this foundation, Zhou et al. [20] integrated the aforementioned models to develop a hybrid model that incorporates imperfect maintenance, thereby substantiating the rationality of the proposed model through numerical illustrations.

However, the aforementioned model fails to incorporate the performance degradation data acquired during the device’s operation, thereby compromising the accuracy of decision optimization. To address this issue, statistical data-driven methods incorporating imperfect maintenance are widely employed. Guo et al. [21] first established a maintenance decision model based on the Wiener process, which introduces a residual degradation model to characterize the impact of imperfect maintenance on degrading devices. To account for the impact of imperfect maintenance on the degradation rate, Zhang et al. [22] introduced a stochastic enhancement factor to establish a novel model that incorporates the effects of imperfect maintenance. Wang et al. [23] proposed a Wiener process model that incorporates negative jumps to predict the device’s RUL during maintenance activities, which solely considers the impact of maintenance activities on the degradation state, without taking into account their influence on the degradation rate. The accurate description of the degradation process for repairable degrading devices necessitates considering the influence of maintenance activities on both the degradation state and rate. Hu et al. [24,25] established a degradation model that incorporates the impact of imperfect maintenance activities on both the rate and state of degradation. Based on this foundation, Ma et al. [26] constructed a multi-phase Wiener-process-based degradation model to characterize the degradation process subjected to imperfect maintenance activities. Pang et al. [27] proposed an adaptive RUL prediction method by employing a multi-stage diffusion process to address the challenge of individual variability among degraded devices with imperfect maintenance.

However, the aforementioned methods typically employ an autoregressive model of order 1, i.e., AR(1), to accomplish the adaptive drift of degradation modeling, which is primarily applicable to scenarios of equispaced monitoring of degraded devices. Furthermore, it is important to note that this model fails to account for the variability in adaptive drift during future degradation processes, which consequently affects the accuracy of RUL predictions [28]. To address this problem, this paper proposes an adaptive-Wiener-process-based method for RUL prediction with imperfect maintenance activities. Firstly, an adaptive Wiener process model is developed to depict the multi-stage degradation process incorporating imperfect maintenance. The PDFs of RUL can be derived based on the concept of first hitting time (FHT) and a convolution operator. Subsequently, taking into account the impact of imperfect maintenance on the degradation state and rate, the maximum likelihood estimation (MLE) method is employed for the offline estimation of model parameters, while Bayesian inference is utilized for online parameter updating. Finally, the effectiveness of the proposed method is verified by simulation examples and case studies of repairable gyroscopes. Compared to the work in [25], the primary innovation of this paper is the implementation of an online updating mechanism for model parameters that explicitly accounts for individual variation. Compared with [27], the main innovation of this paper lies in achieving the adaptive drift of future degradation processes. In summary, the primary contribution of this paper is the integration of the adaptive Wiener model with imperfect maintenance strategies, resulting in a comprehensive composite degradation model that incorporates individual variations and enables online updates of model parameters, thereby enhancing the accuracy of RUL prediction.

The remaining parts of this paper are organized as follows. Section 2 demonstrates the study’s motivation and provides the general framework of the degradation model. In Section 3, we introduce the RUL prediction method incorporating imperfect maintenance. Section 4 provides the main results of model parameter identification and updating. Section 5 validates the proposed method by simulation examples and case studies of repairable gyroscopes. Section 6 summarizes the whole paper.

## 2. Motivation and Degradation Modeling

### 2.1. Motivation

In engineering practice, there are typically two categories of thresholds in PdM activities. The first category is the preventive maintenance threshold $\omega_{p}$, which can be optimized and determined using the maintenance decision model. The second category is the failure threshold ω, which is typically established through the device’s design manuals, industry standards, and expert knowledge. In order to determine appropriate maintenance activities, we can compare the degradation state $X\left(t\right)$ with both the preventive maintenance threshold $\omega_{p}$ and failure threshold ω. When the degradation state is below the preventive maintenance threshold, i.e., $X\left(t\right)<\omega_{p}$, the devices operate normally and require no preventive maintenance. When the degradation state falls within the range between the preventive maintenance threshold and the failure threshold, i.e., $\omega_{p}\leq X\left(t\right)<\omega$, it indicates that the devices require preventive maintenance. When the degradation state $X\left(t\right)$ exceeds the failure threshold ω, device failure will occur, and preventive maintenance needs to involve replacement with spare parts.

**Figure 1:**
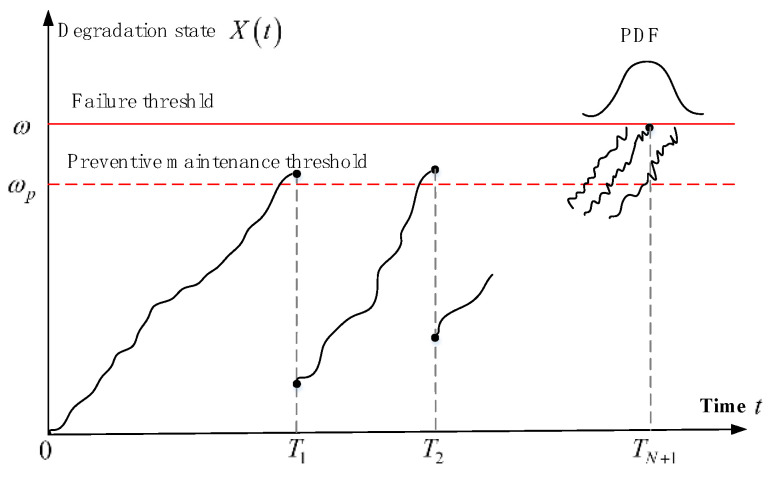
The degradation trajectory in the presence of imperfect maintenance. Where, $\omega_{p}$ and ω represent the preventive maintenance threshold and failure threshold, respectively. $T_{i}$ denotes the time of the *i*th imperfect maintenance activity, while *N* indicates the number of imperfect maintenance activities. It can be observed from Figure 1 that the state and rate of the degradation process are affected by imperfect maintenance.

The cost of preventive maintenance activities is usually influenced by the number of maintenance activities. To ensure the reliability and cost-effectiveness of device operation, maintenance activities are typically constrained in number. Preventive replacement will be implemented instead of preventive maintenance if the degradation state $X\left(t\right)$ exceeds the preventive threshold $\omega_{p}$ after the Nth imperfect maintenance activity. The degradation trajectory for repairable degrading devices is illustrated in Figure 1 [24,25,26,27].

It is worth noting that the degradation rate undergoes changes as the working time continuously accumulates. Nevertheless, existing methods often ignore the adaptive drift variability in future degradation processes, which may affect the accuracy of RUL prediction. Therefore, the focus of this study is to derive the analytical form of the RUL distribution and estimate unknown parameters of the degradation model while considering the impact of imperfect maintenance activities on the degradation state and rate. Based on the actual project requirements, the following reasonable assumptions are made regarding the current situation:

(1) The assumption is made that the same batch of degrading devices consists of M components, with each component functioning independently and exhibiting individual variability in the degradation process. Δt denotes the detection interval, which is usually considered negligible.
(2) The preventive maintenance activities for devices are imperfect maintenance activities, and the number of such activities is limited.
(3) The lifetime and RUL discussed in this study are mainly concerned with the working time of the devices, without taking into account the downtime resulting from preventive maintenance activities.
(4) The degradation processes of components, both prior to and following preventive maintenance activities, are assumed to be statistically independent.

### 2.2. Degradation Model Incorporating Imperfect Maintenance

The degradation model based on the Wiener process is a stochastic model with non-monotonic properties, which has been employed in the fields of RUL prediction and reliability assessment [29,30]. The adaptive Wiener process, as an improved version of the Wiener process, offers significant advantages in dealing with nonuniform measurements and adaptive drift problems. Therefore, it has been widely employed for degradation modeling, aiming to enhance the generality of the degradation model. In particular, an adaptive Wiener process satisfies [28]

(1) $$\begin{array}{l} \lambda \left(t\right)=\lambda_{0}+\kappa W\left(t\right)\\ X(t)=X(0)+\int_{0}^{t}\lambda \left(\tau \right)d\mu \left(\tau ,\theta \right)+\sigma_{B}B(t) \end{array}$$

where $\lambda \left(t\right)$ represents a time-varying drift term that follows a Wiener process. $\lambda_{0}$ denotes the initial drift rate, and κ signifies the diffusion coefficient of the adaptive drift term. $\mu \left(\tau ,\theta \right)$ is a nonlinear function with time t, $W\left(t\right)$ corresponds to a standard Brownian motion independent of $B\left(t\right)$, and $\sigma_{B}$ indicates the diffusion coefficient. If κ=0, the model in Equation (1) can be transformed into a Wiener process model with AR(1).

Under the framework of stochastic degradation modeling, the lifetime T in the concept of FHT can be defined as [31]

(2) T=inf{t:X(t)≥w|X(0)<w)

where w denotes the failure threshold of the devices, and X(0) is the initial degradation state.

Owing to the impact of imperfect maintenance activities, the degradation model of repairable devices is conducted based on the multi-stage Wiener process. When the devices undergo i-1 preventive maintenance activities, the degradation process in the ith stage $X_{i}(t)$ satisfies

(3) $$\left\{\begin{array}{l} \lambda_{i}=\lambda_{i0}+\kappa_{i}W\left(\Delta T_{i}\right)\\ X_{i}(t)=z_{i}+\lambda_{i}\mu \left(\Delta T_{i},\theta \right)+\kappa_{i}\int_{T_{i-1}}^{t}W\left(\tau \right)d\mu \left(\tau ,\theta \right)+\sigma_{B}B\left(\Delta T_{i}\right) \end{array}\right.$$

where $T_{i}$ indicates the time of the ith preventive maintenance activity. 0≤i≤N is the number of maintenance activities performed on the devices before time $T_{i}$, and $\Delta T_{i}=t-T_{i-1}$. N denotes the upper limited number of preventive maintenance activities. $z_{i}$ represents the residual degradation state after i−1 maintenance activities, that is, the initial value of the ith degradation stage. $\lambda_{i}$ represents the drift term of the ith degradation stage, $\lambda_{i0}$ is the initial drift rate, $\kappa_{i}$ is the diffusion coefficient of the adaptive drift term, and $W\left(\cdot \right)$ is a standard Brownian motion independent of $B\left(\cdot \right)$. $\sigma_{B}$ represents the diffusion coefficient of the whole degradation stage. If residual degradation $z_{i}=0$, Equation (3) can be regarded as perfect maintenance; i.e., the special case of imperfect maintenance can still be carried out for parameter identification and RUL prediction.

In this paper, the impacts of preventive maintenance on the degradation state and rate are primarily considered. Specifically, the impact on the degradation state can be represented by the residual degradation state $z_{i}$, while the impact on the degradation rate is denoted as the stage degradation rate $\lambda_{i}$. Firstly, to depict the impact of maintenance activities on the degradation state, the residual degradation state $z_{i}$ is commonly assumed to follow a Gaussian distribution. After i−1 maintenance activities, the probability density function (PDF) of the residual degradation state $z_{i}$ can be defined as [25]

(4) $$f\left(z_{i}\right)=\frac{a^{i-1}b}{1-\exp\left(-ba^{i-1}\right)w_{p}}\times \exp\left[-\frac{a^{i-1}b\left(w_{p}-z_{i}\right)}{w_{p}}\right]I\left(z_{i}\right)$$

where a and b represent hyperparameters. $I\left(z_{i}\right)$ denotes a value function, and

(5) $$I\left(z_{i}\right)=\left\{\begin{array}{l} 1,\;\;\;\;z_{i}\in \left[0,w_{p}\right]\\ 0,\;\;\;\mathrm{else} \end{array}\right.$$

If $z_{i}<0$, it indicates a negative degradation state of the device after maintenance activities, which contradicts the fundamental law governing the degradation process of devices. If $z_{i}=0$, it signifies that the device reverts to a new degradation state following perfect maintenance activities, which deviates from the research in this paper. If $z_{i}\geq \omega_{p}$, it indicates that the degradation state of the component remains unchanged or even exceeds the preventive maintenance threshold after maintenance activities. This means that these maintenance activities lose their significance in ensuring the safe and reliable operation of the devices and extending their service life, which contradicts objective reality.

Subsequently, to accurately depict the influence of maintenance activities on the degradation rate $\lambda_{i}$, a change coefficient of the degradation rate $\zeta_{i}$ was introduced to facilitate subsequent derivation of the RUL distribution under conditions where the degradation model aligns with objective reality, which can be defined by

(6) $$\lambda_{i}=\zeta_{i}\lambda_{1}$$

where $\zeta_{i}$ is the change coefficient after i−1 maintenance activities, i.e., $\zeta_{i}\sim \left(i\mu_{\zeta },\sigma_{\zeta }^{2}\right)$. The degradation rate of the first stage, denoted by $\lambda_{1}$, represents the inherent degradation rate in the absence of any maintenance activities, i.e., $\zeta_{1}=1$. In the 2≤i≤N stage degradation process, the degradation rate of the devices satisfies $\lambda_{i}\sim N\left(\mu_{\lambda_{i}},\sigma_{\lambda_{i}}^{2}\right)$, where $\mu_{\lambda_{i}}=i\mu_{\zeta }\lambda_{10}$ and $\sigma_{\lambda_{i}}^{2}=\sigma_{\zeta }^{2}\lambda_{10}^{2}$.

## 3. RUL Prediction Incorporating Imperfect Maintenance

Let $X_{i,0:t_{k}}=\left\{x_{i,0},x_{i,1},\cdots ,x_{i,t_{k}}\right\}$ represent the condition monitoring (CM) data in the ith degradation stage. According to the definition of FHT, the lifetimes $R_{i,\omega_{P}}$ and $R_{i,\omega }$ when degradation first reaches the thresholds for preventive maintenance and failure can be defined as

(7) $$\begin{array}{c} R_{i,w_{p}}=\inf\left(r_{i,w_{p}}\left|X\left(t_{i,0}+r_{i}\right)\geq w_{p}\right|X\left(t_{i,0}\right)<w_{p}\right)\\ R_{i,w}=\inf\left(r_{i,w}\left|X\left(t_{i,0}+r_{i}\right)\geq w\right|X\left(t_{i,0}\right)<w\right) \end{array}$$

where $r_{i,w_{p}}$ represents the working time after i−1 preventive maintenance activities which the preventive maintenance threshold $\omega_{p}$ is first reached. $r_{i,w}$ represents the working time after i−1 preventive maintenance activities which the failure threshold ω is first reached. Considering the individual variability in the same batch of devices, the adaptive drift term $\lambda_{i}$ is randomized and satisfies $\lambda_{i}\sim N\left(\mu_{\lambda_{i}},\sigma_{\lambda_{i}}^{2}\right)$.

Owing to the inherent stochastic nature of Brownian motion, the lifetime $R_{i,\omega }$ can be identified as a random variable that follows an inverse Gaussian distribution. Thus, the conditional PDF of the predicted lifetime can be expressed as

(8) $$\begin{array}{l} f_{R_{i,\omega }|\lambda_{i}}(r_{i,\omega }|\lambda_{i})=\frac{1}{\sqrt{2\pi \varphi (r_{i,\omega })}}\times \exp\left\{-\frac{{\left[\omega -\int_{0}^{r_{i,\omega }}\lambda_{i}d\mu \left(\tau ,\theta \right)\right]}^{2}}{2\varphi (r_{i,\omega })}\right\}\\ \left[\frac{\omega -\int_{0}^{r_{i,\omega }}\lambda_{i}d\mu \left(\tau ,\theta \right)}{\varphi (r_{i,\omega })}\varphi^{\prime }(r_{i,\omega })+\lambda_{i}\mu^{\prime }\left(r_{i,\omega },\theta \right)\right]\\ \varphi (r_{i,\omega })=\kappa^{2}\int_{0}^{r_{i,\omega }}{\left(\mu \left(r_{i,\omega },\theta \right)-\mu \left(\tau ,\theta \right)\right)}^{2}d\tau +\sigma_{B}^{2}r_{i,\omega }\\ \varphi^{\prime }(r_{i,\omega })=d\varphi (r_{i,\omega })/dr_{i,\omega }=2\kappa^{2}\int_{0}^{r_{i,\omega }}\left(\mu \left(r_{i,\omega },\theta \right)-\mu \left(\tau ,\theta \right)\right)d\tau +\sigma_{B}^{2} \end{array}$$

To consider the individual variation in devices, we introduce Lemma 1, as presented in our previous work [32], to tackle the aforementioned integral.

**Lemma** **1** [32]. *If $p\sim N(u,\sigma^{2})$, A,B∈ℝ, and $C\in {\mathbb{R}}^{+}$, then the following formula holds:*

(9) $$E_{p}\left[(A-p)\exp\left(-\frac{{\left(B-p\right)}^{2}}{2C}\right)\right]=\sqrt{\frac{C}{\sigma^{2}+C}}\left(A-\frac{\sigma^{2}B+uC}{\sigma^{2}+C}\right)\cdot \exp\left(-\frac{{\left(B-u\right)}^{2}}{2(\sigma^{2}+C)}\right)$$

Therefore, owing to Lemma 1 and the full probability formula, the PDF and cumulative density function (CDF) for lifetimes $R_{i,\omega_{P}}$ and $R_{i,\omega }$ that consider the influence of imperfect maintenance activities can be derived, respectively:

(10) $$\begin{array}{l} f_{R_{i,\omega_{p}}}\left(r_{i,\omega_{p}}|z_{i}\right)=\frac{1}{\sqrt{2\pi \left(\varphi (r_{i,\omega_{p}})+\sigma_{\lambda_{i}}^{2}\mu^{2}\left(r_{i,\omega_{p}},\theta \right)\right)}}\times \exp\left\{-\frac{{\left(\omega_{p}-z_{i}-\mu_{\lambda_{i}}\mu \left(r_{i,\omega_{p}},\theta \right)\right)}^{2}}{2\left(\varphi (r_{i,\omega_{p}})+\sigma_{\lambda_{i}}^{2}\mu^{2}\left(r_{i,\omega_{p}},\theta \right)\right)}\right\}\\ \left(\frac{\varphi^{\prime }(r_{i,\omega_{p}})}{\varphi (r_{i,\omega_{p}})}\left(\omega_{p}-z_{i}\right)+\left(\mu^{\prime }\left(r_{i,\omega_{p}},\theta \right)-\frac{\varphi^{\prime }(r_{i,\omega_{p}})}{\varphi (r_{i,\omega_{p}})}\mu \left(r_{i,\omega_{p}},\theta \right)\right)\frac{\varphi (r_{i,\omega_{p}})\mu_{\lambda_{i}}+\left(\omega_{p}-z_{i}\right)\sigma_{\lambda_{i}}^{2}\mu \left(r_{i,\omega_{p}},\theta \right)}{\varphi (r_{i,\omega_{p}})+\sigma_{\lambda i}^{2}\mu^{2}\left(r_{i,\omega_{p}},\theta \right)}\right)\\ \mu_{\lambda_{i}}=i\mu_{\zeta }\lambda_{10},\sigma_{\lambda_{i}}^{2}=\sigma_{\zeta }^{2}\lambda_{10}^{2}\\ \varphi (r_{i,\omega_{p}})=\kappa^{2}\int_{0}^{r_{i,\omega_{p}}}{\left(\mu \left(r_{i,\omega_{p}},\theta \right)-\mu \left(\tau ,\theta \right)\right)}^{2}d\tau +\sigma_{B}^{2}r_{i,\omega_{p}}\\ \varphi^{\prime }(r_{i,\omega_{p}})=\frac{d\varphi (r_{i,\omega_{p}})}{dr_{i,\omega_{p}}}=2\kappa^{2}\int_{0}^{r_{i,\omega_{p}}}\left(\mu \left(r_{i,\omega_{p}},\theta \right)-\mu \left(\tau ,\theta \right)\right)d\tau +\sigma_{B}^{2} \end{array}$$

(11) $$\begin{array}{cc} F_{i,w_{p}}\left(r_{i,w_{p}}|z_{i}\right) & =P\left\{R_{i,w_{p}}<r_{i}|z_{i}\right\}\\ & =\Phi \left(\frac{-\left(w_{p}-z_{i}\right)+\mu_{\lambda_{i}}\mu \left(r_{i,\omega_{p}},\theta \right)}{\sqrt{\varphi^{\prime }(r_{i,\omega_{p}})+\sigma_{\lambda_{i}}^{2}\mu^{2}\left(r_{i,\omega_{p}},\theta \right)}}\right)+\exp\left(\frac{2\mu_{\lambda_{i}}\left(w_{p}-z_{i}\right)}{\varphi^{\prime }(r_{i,\omega_{p}})}+\frac{2\sigma_{\lambda_{i}}^{2}\left(w_{p}-z_{i}\right)}{\varphi^{\prime }{\left(r_{i,\omega_{p}}\right)}^{2}}\right)\\ & \times \Phi \left(-\frac{2\sigma_{\lambda_{i}}^{2}\left(w_{p}-z_{i}\right)\mu \left(r_{i,\omega_{p}},\theta \right)+\varphi^{\prime }{\left(r_{i,\omega_{p}}\right)}^{2}\left(\mu_{\lambda_{i}}\mu \left(r_{i,\omega_{p}},\theta \right)+w_{p}\right)}{\varphi^{\prime }(r_{i,\omega_{p}})\sqrt{\varphi (r_{i,\omega_{p}})+\sigma_{\lambda_{i}}^{2}\mu^{2}\left(r_{i,\omega_{p}},\theta \right)}}\right) \end{array}$$

Furthermore, the PDF and CDF for lifetime $R_{i,\omega }$ exhibit similar mathematical forms. On the basis of deducing the lifetime distribution in the i−1 th stage of imperfect maintenance, it can be observed from the previous assumptions that when considering the impact of imperfect maintenance, the lifetime mainly consists of two parts. One is the cumulative working time in stage 1≤i≤N, which refers to the duration until the degradation state reaches the preventive maintenance threshold for the first time after each imperfect maintenance activity. The other one is the working time when the degradation state first reaches the failure threshold in stage N+1. In this case, the whole lifetime T can be expressed as follows:

(12) $$T=\sum_{i=1}^{N}R_{i,\omega_{P}}+R_{N+1,\omega }$$

When the degradation state $X_{i,k}$ at time $t_{i,k}$ is provided in the ith stage, the corresponding RUL can be defined as

(13) $$R_{i,k}=\inf\left(r_{i,k}\left|X\left(t_{i,k}+r_{i}\right)\geq w_{p}\right|X\left(t_{i,k}\right)<w_{p}\right)$$

where $f_{R_{i,k}|X_{i,0:k}}\left(r_{i,k}|X_{i,0:k}\right)$ denotes the PDF of stage RUL at time $t_{i,k}$.

Similarly, the whole RUL corresponding to the component at time $t_{i,k}$ satisfies

(14) $$L_{i,k}=R_{i,k}+I(i)\sum_{m=1}^{N-i+1}R_{i+m}$$

where I(i) is the value function, satisfying

(15) $$I(i)=\left\{\begin{array}{l} 1,\;\;1\leq i\leq N\\ 0,\;\;i=N+1 \end{array}\right.$$

Let $f_{L_{i,k}|X_{1:i,0:k}}\left(l_{i,k}|X_{1:i,0:k}\right)$ denote the PDF of the whole RUL at time $t_{i,k}$. According to the knowledge of probability theory and the convolution operator, the PDF of the device’s RUL incorporating imperfect maintenance can be specifically expressed as follows:

(16) $$\left\{\begin{array}{l} f_{L_{i,k}|X_{1:i,0:k}}\left(l_{i,j}|X_{1:i,0:k}\right)=f_{L_{N+1,k}|X_{N+1,0;k}}\left(l_{N+1,k}|X_{N+1,0:k}\right),\;\;\;\;i=N+1\\ f_{L_{i,k}|X_{1:i,0:k}}\left(l_{i,j}|X_{1:i,0:k}\right)=f_{R_{i,k}|X_{i,0:k}}\left(r_{i,k}|X_{i,0:k}\right)⊗f_{R_{i+1,\omega_{p}}|X_{i+1,0:K_{i+1}}}\left(r_{i+1,\omega_{p}}|X_{i+1,0:K_{i+1}}\right)\\ ⊗\cdots ⊗f_{R_{N+1},\omega |X_{N+1,0:K_{N+1}}}\left(r_{N+1,\omega }|X_{N+1,0:K_{N+1}}\right),\;\;\;\;1⩽i⩽N \end{array}\right.$$

where ⊗ represents the convolution symbol. Based on the aforementioned analysis, the RUL prediction of degrading devices incorporating imperfect maintenance can be divided into two cases, i.e., i=N+1 and 1≤i≤N.

(1). If i=N+1, it indicates that the device has undergone Nth imperfect maintenance activities. In this case, the stage RUL in the N+1 th stage $R_{N+1,k}$ is equivalent to the whole RUL of devices $L_{N+1,k}$, and the PDFs of RUL can be further expressed as

(17) $$\begin{array}{l} f_{L_{N+1,k}|\lambda_{N+1,k}}\left(l_{N+1,k}|\lambda_{N+1,k}\right)\\ =\frac{1}{\sqrt{2\pi \left(\varphi (l_{N+1,k})+\sigma_{\lambda_{N+1}}^{2}\mu^{2}\left(l_{N+1,k},\theta \right)\right)}}\exp\left\{-\frac{{\left(\omega -x_{N+1,k}-\mu_{\lambda_{N+1}}\mu \left(l_{N+1,k},\theta \right)\right)}^{2}}{2\left(\varphi (l_{N+1,k})+\sigma_{\lambda_{N+1}}^{2}\mu^{2}\left(l_{N+1,k},\theta \right)\right)}\right\}\\ \times \left(\begin{array}{l} \frac{\varphi^{\prime }(l_{N+1,k})}{\varphi (l_{N+1,k})}\left(\omega -x_{N+1,k}\right)+\left(\mu^{\prime }\left(l_{N+1,k},\theta \right)-\frac{\varphi^{\prime }(l_{N+1,k})}{\varphi (l_{N+1,k})}\mu \left(l_{N+1,k},\theta \right)\right)\\ \frac{\varphi (l_{N+1,k})\mu_{\lambda_{N+1}}+\left(\omega -x_{N+1,k}\right)\sigma_{\lambda_{N+1}}^{2}\mu \left(l_{N+1,k},\theta \right)}{\varphi (l_{N+1,k})+\sigma_{\lambda_{N+1}}^{2}\mu^{2}\left(l_{N+1,k},\theta \right)} \end{array}\right)\\ \mu_{\lambda_{N+1}}=\left(N+1\right)\mu_{\zeta }\lambda_{10},\sigma_{\lambda_{N+1}}^{2}=\sigma_{\zeta }^{2}\lambda_{10}^{2}\\ \varphi (l_{N+1,k})=\kappa^{2}\int_{0}^{l_{N+1,k}}{\left[\mu \left(l_{N+1,k},\theta \right)-\mu \left(\tau ,\theta \right)\right]}^{2}d\tau +\sigma_{B}^{2}l_{N+1,k}\\ \varphi^{\prime }(l_{N+1,k})=\frac{d\varphi (l_{N+1,k})}{dl_{N+1,k}}=2\kappa^{2}\int_{0}^{l_{N+1,k}}\left[\mu \left(l_{N+1,k},\theta \right)-\mu \left(\tau ,\theta \right)\right]d\tau +\sigma_{B}^{2} \end{array}$$

(2). If 1≤i≤N, $f_{R_{i,k}|\lambda_{i,k}}\left(r_{i,k}|\lambda_{i,k}\right)$, $f_{R_{i+j}|\lambda_{i+j,k}}\left(r_{i+j}|\lambda_{i+j,k}\right)$, and $f_{R_{N+1}|\lambda_{N+1,k}}\left(r_{N+1}|\lambda_{N+1,k}\right)$ represent the PDF of the stage RUL at time $t_{i,k}$, the PDF of operational time $R_{i+j}$ in the $i+j\;\left(1\leq j\leq N-i\right)$ th stage, and the PDF of operational time in the N+1 th stage, respectively. Based on the RUL distribution in an adaptive Wiener process, the PDFs of RUL can be derived as

(18) $$\begin{array}{l} f_{R_{i,k}|\lambda_{i,k}}\left(r_{i,k}|\lambda_{i,k}\right)=\frac{1}{\sqrt{2\pi \left(\varphi (r_{i,k})+\sigma_{\lambda_{i}}^{2}\mu^{2}\left(r_{i,k},\theta \right)\right)}}\times \exp\left\{-\frac{{\left(\omega_{p}-x_{i,k}-\mu_{\lambda_{i}}\mu \left(r_{i,k},\theta \right)\right)}^{2}}{2\left(\varphi (r_{i,k})+\sigma_{\lambda_{i}}^{2}\mu^{2}\left(r_{i,k},\theta \right)\right)}\right\}\\ \left(\frac{\varphi^{\prime }(r_{i,k})}{\varphi (r_{i,k})}\left(\omega_{p}-x_{i,k}\right)+\left(\mu^{\prime }\left(r_{i,k},\theta \right)-\frac{\varphi^{\prime }(r_{i,k})}{\varphi (r_{i,k})}\mu \left(r_{i,k},\theta \right)\right)\frac{\varphi (r_{i,k})\mu_{\lambda_{i}}+\left(\omega -x_{i,k}\right)\sigma_{\lambda_{N+1}}^{2}\mu \left(r_{i,k},\theta \right)}{\varphi (r_{i,k})+\sigma_{\lambda_{i}}^{2}\mu^{2}\left(r_{i,k},\theta \right)}\right)\\ \mu_{\lambda_{i}}=i\mu_{\zeta }\lambda_{10},\sigma_{\lambda_{i}}^{2}=\sigma_{\zeta }^{2}\lambda_{10}^{2}\\ \varphi (r_{i,k})=\kappa^{2}\int_{0}^{r_{i,k}}{\left[\mu \left(r_{i,k},\theta \right)-\mu \left(\tau ,\theta \right)\right]}^{2}d\tau +\sigma_{B}^{2}r_{i,k}\\ \varphi^{\prime }(r_{i,k})=\frac{d\varphi (r_{i,k})}{dr_{i,k}}=2\kappa^{2}\int_{0}^{r_{i,k}}\left[\mu \left(r_{i,k},\theta \right)-\mu \left(\tau ,\theta \right)\right]d\tau +\sigma_{B}^{2} \end{array}$$

(19) $$\begin{array}{l} f_{R_{i+j}|\lambda_{i+j,k}}\left(r_{i+j}|\lambda_{i+j,k}\right)=\frac{1}{\sqrt{2\pi \left(\varphi (r_{i+j})+\sigma_{\lambda_{i+j}}^{2}\mu^{2}\left(r_{i+j},\theta \right)\right)}}\times \exp\left\{-\frac{{\left(\omega_{p}-z_{i+j}-\mu_{\lambda_{i+j}}\mu \left(r_{i+j},\theta \right)\right)}^{2}}{2\left(\varphi (r_{i+j})+\sigma_{\lambda_{i+j}}^{2}\mu^{2}\left(r_{i+j},\theta \right)\right)}\right\}\\ \left(\frac{\varphi^{\prime }(r_{i+j})}{\varphi (r_{i+j})}\left(\omega_{p}-z_{i+j}\right)+\left(\mu^{\prime }\left(r_{i+j},\theta \right)-\frac{\varphi^{\prime }(r_{i+j})}{\varphi (r_{i+j})}\mu \left(r_{i+j},\theta \right)\right)\frac{\varphi (r_{i+j})\mu_{\lambda_{i}+j}+\left(\omega -z_{i+j}\right)\sigma_{\lambda_{i+j}}^{2}\mu \left(r_{i+j},\theta \right)}{\varphi (r_{i+j})+\sigma_{\lambda_{i+j}}^{2}\mu^{2}\left(r_{i+j},\theta \right)}\right)\\ \mu_{\lambda_{i+j}}=\left(i+j\right)\mu_{\zeta }\lambda_{10},\sigma_{\lambda_{i+j}}^{2}=\sigma_{\zeta }^{2}\kappa^{2}r_{i+j}\\ \varphi (r_{i+j})=\kappa^{2}\int_{0}^{r_{i+j}}{\left[\mu \left(r_{i+j},\theta \right)-\mu \left(\tau ,\theta \right)\right]}^{2}d\tau +\sigma_{B}^{2}r_{i+j}\\ \varphi^{\prime }(r_{i+j})=\frac{d\varphi (r_{i+j})}{dr_{i+j}}=2\kappa^{2}\int_{0}^{r_{i+j}}\left[\mu \left(r_{i+j},\theta \right)-\mu \left(\tau ,\theta \right)\right]d\tau +\sigma_{B}^{2} \end{array}$$

(20) $$\begin{array}{l} f_{R_{N+1}|\lambda_{N+1,k}}\left(r_{N+1}|\lambda_{N+1,k}\right)=\frac{1}{\sqrt{2\pi \left(\varphi (r_{N+1})+\sigma_{\lambda_{N+1}}^{2}\mu^{2}\left(r_{N+1},\theta \right)\right)}}\times \exp\left\{-\frac{{\left(\omega -z_{N+1}-\mu_{\lambda_{N+1}}\mu \left(r_{N+1},\theta \right)\right)}^{2}}{2\left(\varphi (r_{N+1})+\sigma_{\lambda_{N+1}}^{2}\mu^{2}\left(r_{N+1},\theta \right)\right)}\right\}\\ \left(\frac{\varphi^{\prime }(r_{N+1})}{\varphi (r_{N+1})}\left(\omega -z_{N+1}\right)+\left(\mu^{\prime }\left(r_{N+1},\theta \right)-\frac{\varphi^{\prime }(r_{N+1})}{\varphi (r_{N+1})}\mu \left(r_{N+1},\theta \right)\right)\frac{\varphi (r_{N+1})\mu_{\lambda_{N+1}}+\left(\omega -z_{N+1}\right)\sigma_{\lambda_{N+1}}^{2}\mu \left(r_{N+1},\theta \right)}{\varphi (r_{N+1})+\sigma_{\lambda_{N+1}}^{2}\mu^{2}\left(r_{N+1},\theta \right)}\right)\\ \mu_{\lambda_{N+1}}=\left(N+1\right)\mu_{\zeta }\lambda_{10},\sigma_{\lambda_{N+1}}^{2}=\sigma_{\zeta }^{2}\lambda_{10}^{2}\\ \varphi (r_{N+1})=\kappa^{2}\int_{0}^{r_{N+1}}{\left[\mu \left(r_{N+1},\theta \right)-\mu \left(\tau ,\theta \right)\right]}^{2}d\tau +\sigma_{B}^{2}r_{N+1}\\ \varphi^{\prime }(r_{N+1})=\frac{d\varphi (r_{N+1})}{dr_{N+1}}=2\kappa^{2}\int_{0}^{r_{N+1}}\left[\mu \left(r_{N+1},\theta \right)-\mu \left(\tau ,\theta \right)\right]d\tau +\sigma_{B}^{2} \end{array}$$

## 4. Model Parameter Identification and Updating

Based on the degradation model (3) incorporating imperfect maintenance, the estimated parameters are denoted by $\Theta =\left\{a,b,\lambda_{10},\mu_{\zeta ,}\sigma_{\zeta }^{2},\kappa ,\sigma_{B}^{2},\theta \right\}$, where $\Theta_{1}=\left\{a,b\right\}$ represents the hyperparameters of the residual degradation state $z_{i}$, and $\Theta_{2}=\left\{\lambda_{10},\mu_{\zeta ,}\sigma_{\zeta }^{2},\kappa ,\sigma_{B}^{2},\theta \right\}$ denotes the unknown parameters of the operational degradation model.

### 4.1. Parameter Estimation of Residual Degradation State

The estimation of residual degradation hyperparameters $\Theta_{1}=\left\{a,b\right\}$ requires the collection of historical data on residual degradation after each imperfect maintenance activity. Assume that we have gathered the CM data from M devices and that each device has undergone N imperfect maintenance activities. Subsequently, let $Z_{1:N}^{j}=\left\{z_{1}^{j},z_{2}^{j},\cdots z_{N}^{j}\right\}$ denote the historical residual degradation data of the jth device, where 1≤j≤M, $j\in {\mathbb{N}}^{+}$.

According to the definition of the residual degradation state in (4), the residual degradation coefficient of the ith stage can be expressed as

(21) $$\gamma_{i}^{j}=z_{i}^{j}/\omega_{p}$$

where 1≤i≤N, and $\gamma_{1:N}^{j}=\left\{\gamma_{1}^{j},\gamma_{2}^{j},\cdots \gamma_{N}^{j}\right\}$ represents the residual coefficient of the jth device. Furthermore, the hyperparameter $\Theta_{1}=\left\{a,b\right\}$ of the residual degradation state can be estimated utilizing the MLE algorithm, and its likelihood function can be presented as

(22) $${\hat{\Theta }}_{1}=\underset{\Theta_{1}}{\arg\max}l\left(\Theta_{1}\right)=\underset{\Theta_{1}}{\arg\max}\left[-\frac{MN}{2}\ln2\pi -\frac{MN}{2}\ln b-\frac{\sum_{j=1}^{M}\sum_{i=1}^{N}{\left(\gamma_{i}^{j}-(1-\exp(-ai))\right)}^{2}}{2b}\right]$$

where $l\left(\Theta_{1}\right)$ represents the log-likelihood function. The relationship between a and b can be derived by maximizing the likelihood function in (22).

(23) $$b=\frac{1}{MN}\sum_{j=1}^{M}\sum_{i=1}^{N}{\left(\gamma_{i}^{j}-(1-\exp(-ai))\right)}^{2}$$

Then, substituting the estimated value of b into Equation (23) can provide the maximum likelihood estimation result of parameter a.

### 4.2. Parameter Estimation of Degradation Model

The parameters $\Theta_{2}=\left\{\lambda_{10},\mu_{\zeta ,}\sigma_{\zeta }^{2},\kappa ,\sigma_{B}^{2},\theta \right\}$ can be estimated through the historical degradation data of repairable degrading devices. Let $X_{0:N,k}^{j}=\left\{x_{0,0}^{j},x_{0,1}^{j},\cdots x_{N,k-1}^{j},x_{N,k}^{j}\right\}$ denote the CM data of the jth device. Owing to the impact of the change coefficient $\zeta_{i}\sim N\left(i\mu_{\zeta },\sigma_{\zeta }^{2}\right)$, the estimation of model parameters needs to be divided into two cases for analysis, i.e., i=1 and 2≤i≤N+1.

(1). If i=1, based on the degradation model (3) and the characteristics of the diffusion process, $X_{0:N,k}^{j}$ is assumed to follow a multidimensional normal distribution, and its mean and covariance should be expressed.

(24) $$\begin{array}{l} \left\{x_{1,k}^{j}|x_{1,k-1}^{j},\lambda_{10},\kappa ,\sigma_{B}^{2},\theta \right\}∼\\ N\left(x_{1,k-1}^{j}+\lambda_{10}\mu \left(t_{1,k}^{j}-t_{1,k-1}^{j},\theta \right),\kappa^{2}\int_{0}^{t_{1,k}^{j}-t_{1,k-1}^{j}}{\left[\mu \left(t_{1,k}^{j}-t_{1,k-1}^{j},\theta \right)-\mu \left(\tau ,\theta \right)\right]}^{2}d\tau +\sigma_{B}^{2}\left(t_{1,k}^{j}-t_{1,k-1}^{j}\right)\right) \end{array}$$

Therefore, the conditional probability distribution of $\left\{x_{1,k}^{j}|x_{1,k-1}^{j},\lambda_{10},\kappa ,\sigma_{B}^{2},\theta \right\}$ can be expressed as

(25) $$\begin{array}{cc} p\left(x_{1,k}^{j}|x_{1,k-1}^{j},\lambda_{10},\sigma_{B}^{2},\kappa \right) & =\frac{1}{\sqrt{2\pi \varphi \left(t_{1,k}^{j}-t_{1,k-1}^{j}\right)}}\left[\begin{array}{l} \frac{x_{1,k}^{j}-x_{1,k-1}^{j}-\lambda_{10}\mu \left(t_{1,k}^{j}-t_{1,k-1}^{j},\theta \right)}{\varphi \left(t_{1,k}^{j}-t_{1,k-1}^{j}\right)}\\ \times \varphi^{\prime }\left(t_{1,k}^{j}-t_{1,k-1}^{j}\right)+\lambda_{10}\mu^{\prime }\left(t_{1,k}^{j}-t_{1,k-1}^{j},\theta \right) \end{array}\right]\\ & \times \exp\left\{-\frac{{\left[x_{1,k}^{j}-x_{1,k-1}^{j}-\lambda_{10}\mu \left(t_{1,k}^{j}-t_{1,k-1}^{j},\theta \right)\right]}^{2}}{2\varphi \left(t_{1,k}^{j}-t_{1,k-1}^{j}\right)}\right\} \end{array}$$

where $\begin{array}{l} \varphi (t_{1,k}^{j}-t_{1,k-1}^{j})=\kappa^{2}\int_{0}^{t_{1,k}^{j}-t_{1,k-1}^{j}}{\left[\mu \left(t_{1,k}^{j}-t_{1,k-1}^{j},\theta \right)-\mu \left(\tau ,\theta \right)\right]}^{2}d\tau +\sigma_{B}^{2}\left(t_{1,k}^{j}-t_{1,k-1}^{j}\right)\\ \varphi^{\prime }(t_{1,k}^{j}-t_{1,k-1}^{j})=\frac{d\varphi (t_{1,k}^{j}-t_{1,k-1}^{j})}{d\left(t_{1,k}^{j}-t_{1,k-1}^{j}\right)}=2\kappa^{2}\int_{0}^{t_{1,k}^{j}-t_{1,k-1}^{j}}\left[\mu \left(t_{1,k}^{j}-t_{1,k-1}^{j},\theta \right)-\mu \left(\tau ,\theta \right)\right]d\tau +\sigma_{B}^{2} \end{array}$.

Based on the chain rule and the Markov property, the likelihood function of the degradation model can be derived from the CM data of the first stage as follows:

(26) $$\begin{array}{cc} L\left(\lambda_{10},\sigma_{B}^{2},\kappa ,\theta \right) & =\prod_{j=1}^{M}\prod_{k=1}^{k_{i}}\frac{1}{\sqrt{2\pi \varphi \left(t_{1,k}^{j}-t_{1,k-1}^{j}\right)}}\left[\begin{array}{l} \frac{x_{1,k}^{j}-x_{1,k-1}^{j}-\lambda_{10}\mu \left(t_{1,k}^{j}-t_{1,k-1}^{j},\theta \right)}{\varphi \left(t_{1,k}^{j}-t_{1,k-1}^{j}\right)}\\ \times \varphi^{\prime }\left(t_{1,k}^{j}-t_{1,k-1}^{j}\right)+\lambda_{10}\mu^{\prime }\left(t_{1,k}^{j}-t_{1,k-1}^{j},\theta \right) \end{array}\right]\\ & \times \exp\left\{-\frac{{\left[x_{1,k}^{j}-x_{1,k-1}^{j}-\lambda_{10}\mu \left(t_{1,k}^{j}-t_{1,k-1}^{j},\theta \right)\right]}^{2}}{2\varphi \left(t_{1,k}^{j}-t_{1,k-1}^{j}\right)}\right\} \end{array}$$

(2). If 2≤i≤N+1, based on $\lambda_{i}=\zeta_{i}\lambda_{1}$ and $\zeta_{i}\sim N\left(i\mu_{\xi },\sigma_{\zeta }^{2}\right)$, the conditional probability distribution of $\left\{x_{i,k}^{j}|x_{i,k-1}^{j},\lambda_{10},\mu_{\zeta ,}\sigma_{\zeta }^{2},\kappa ,\sigma_{B}^{2}\right\}$ can be obtained.

(27) $$\begin{array}{c} p\left(x_{i,k}^{j}|x_{i,k-1}^{j},\lambda_{10},\mu_{\zeta ,}\sigma_{\zeta }^{2},\kappa ,\sigma_{B}^{2}\right)\\ =\frac{1}{\sqrt{2\pi G}}\left[\frac{x_{i,k}^{j}-x_{i,k-1}^{j}-i\mu_{\zeta }\lambda_{10}\mu \left(t_{i,k}^{j}-t_{i,k-1}^{j},\theta \right)}{G}\varphi^{\prime }\left(t_{i,k}^{j}-t_{i,k-1}^{j}\right)+i\mu_{\zeta }\lambda_{10}\mu^{\prime }\left(t_{i,k}^{j}-t_{i,k-1}^{j},\theta \right)\right]\\ \times \exp\left\{-\frac{{\left[x_{i,k}^{j}-x_{i,k-1}^{j}-i\mu_{\zeta }\lambda_{10}\mu \left(t_{i,k}^{j}-t_{i,k-1}^{j},\theta \right)\right]}^{2}}{2G}\right\} \end{array}$$

where $\begin{array}{l} G=\kappa^{2}\int_{0}^{t_{i,k}^{j}-t_{i,k-1}^{j}}{\left[\mu \left(t_{i,k}^{j}-t_{i,k-1}^{j},\theta \right)-\mu \left(\tau ,\theta \right)\right]}^{2}d\tau +\sigma_{B}^{2}\left(t_{i,k}^{j}-t_{i,k-1}^{j}\right)+\sigma_{\zeta }^{2}\int_{t_{i,k-1}^{j}}^{t_{i,k}^{j}}\lambda_{10}d\mu \left(\tau ,\theta \right)\\ \varphi^{\prime }(t_{i,k}^{j}-t_{i,k-1}^{j})=2\kappa^{2}\int_{0}^{t_{i,k}^{j}-t_{i,k-1}^{j}}\left[\mu \left(t_{i,k}^{j}-t_{i,k-1}^{j},\theta \right)-\mu \left(\tau ,\theta \right)\right]d\tau +\sigma_{B}^{2} \end{array}$.

Similarly, based on the chain rule and the Markov property, the likelihood function of the degradation model can be derived from the CM data of the ith stage as follows:

(28) $$\begin{array}{c} L_{i}\left(\lambda_{10},\mu_{\zeta ,}\sigma_{\zeta }^{2},\kappa ,\sigma_{B}^{2}\right)\\ =\prod_{j=1}^{M}\prod_{k=1}^{k_{i}}\frac{1}{\sqrt{2\pi G}}\left[\frac{x_{i,k}^{j}-x_{i,k-1}^{j}-i\mu_{\zeta }\lambda_{10}\mu \left(t_{i,k}^{j}-t_{i,k-1}^{j},\theta \right)}{G}\varphi^{\prime }\left(t_{i,k}^{j}-t_{i,k-1}^{j}\right)+i\mu_{\zeta }\lambda_{10}\mu^{\prime }\left(t_{i,k}^{j}-t_{i,k-1}^{j},\theta \right)\right]\\ \times \exp\left\{-\frac{{\left[x_{i,k}^{j}-x_{i,k-1}^{j}-i\mu_{\zeta }\lambda_{10}\mu \left(t_{i,k}^{j}-t_{i,k-1}^{j},\theta \right)\right]}^{2}}{2G}\right\} \end{array}$$

According to the assumed condition, the degradation processes of devices at each stage are assumed to be mutually independent. Therefore, the likelihood function can be further constructed based on historical degradation data from M devices within the same batch.

(29) $$L\left(\lambda_{10},\mu_{\zeta ,}\sigma_{\zeta }^{2},\kappa ,\sigma_{B}^{2},\theta \right)=L\left(\lambda_{10},\sigma_{B}^{2},\kappa ,\theta \right)\prod_{i=2}^{N+1}L_{i}\left(\lambda_{10},\mu_{\zeta ,}\sigma_{\zeta }^{2},\kappa ,\sigma_{B}^{2},\theta \right)$$

By maximizing the aforementioned Equation (29), we can obtain the maximum likelihood estimation of the unknown parameters $\Theta_{2}=\left\{\lambda_{10},\mu_{\zeta ,}\sigma_{\zeta }^{2},\kappa ,\sigma_{B}^{2},\theta \right\}$ in the degradation model.

In addition, considering the random characteristics of the parameter $\zeta_{i}$, after experiencing i instances of imperfect maintenance, the degradation data $X_{i,0:k}=\left\{x_{i,0},x_{i,1},\cdots x_{i,k-1},x_{i,k}\right\}$ can be obtained at the current time $t_{i,k}$. Owing to the imperfect degradation model established in Equation (3) and the Bayesian chain rule, the joint probability density function of the degradation data $X_{i,0:k}$ can be derived as follows:

(30) $$\begin{array}{c} p\left(X_{i,0:k}|\zeta_{i}\right)=\prod_{j=1}^{k}\frac{1}{\sqrt{2\pi \varphi \left(\Delta \right)}}\cdot \exp\left(-\frac{{\left(X\left(t_{i,j}\right)-X\left(t_{i,j-1}\right)-\zeta_{i}\lambda_{10}\Delta \right)}^{2}}{2\varphi \left(\Delta \right)}\right)\\ \left[\frac{\left(X\left(t_{i,j}\right)-X\left(t_{i,j-1}\right)-\zeta_{i}\lambda_{10}\Delta \right)}{\varphi (\Delta )}\varphi^{\prime }(\Delta )+\zeta_{i}\lambda_{10}\Delta^{\prime }\right] \end{array}$$

where $\Delta =\mu \left(t_{i,j}-t_{i,j-1},\theta \right)$. Let the prior distribution of $\zeta_{i}$ be denoted by $p\left(\zeta_{i}\right)$, and $\zeta_{i}\sim \left(i\mu_{\zeta ,i0},\sigma_{\zeta ,i0}^{2}\right)$ can be obtained by parameter updating after i−1 imperfect maintenance activities. Based on the Bayesian theorem, the posterior distribution of the random parameter $\zeta_{i}$ can be calculated as follows:

(31) $$p\left(\zeta_{i}|X_{i,0:k}\right)\propto p\left(X_{i,0:k}|\zeta_{i}\right)p\left(\zeta_{i}\right)=p\left(X_{i,0:k}|\zeta_{i}\right)\cdot \frac{1}{\sqrt{2\pi \sigma_{\zeta ,i0}^{2}}}\cdot \exp\left(-\frac{{\left(\zeta_{i}-i\mu_{\zeta ,i0}\right)}^{2}}{2\sigma_{\zeta ,i0}^{2}}\right)$$

## 5. Case Study

In this section, simulation cases and degradation data of repairable gyroscopes are employed to verify the validity of the proposed model. For the sake of experimental convenience, we designate the proposed method as M0, while we refer to the imperfect maintenance degradation model presented in previous work [25,27] as M1 and the degradation model that neglects the influence of imperfect maintenance discussed in [29] as M2. Although method M1 incorporates the impact of preventive maintenance activities on the device’s degradation state and rate, it often fails to account for the variability in future degradation caused by adaptive drift.

### 5.1. Numerical Simulation

**Figure 2:**
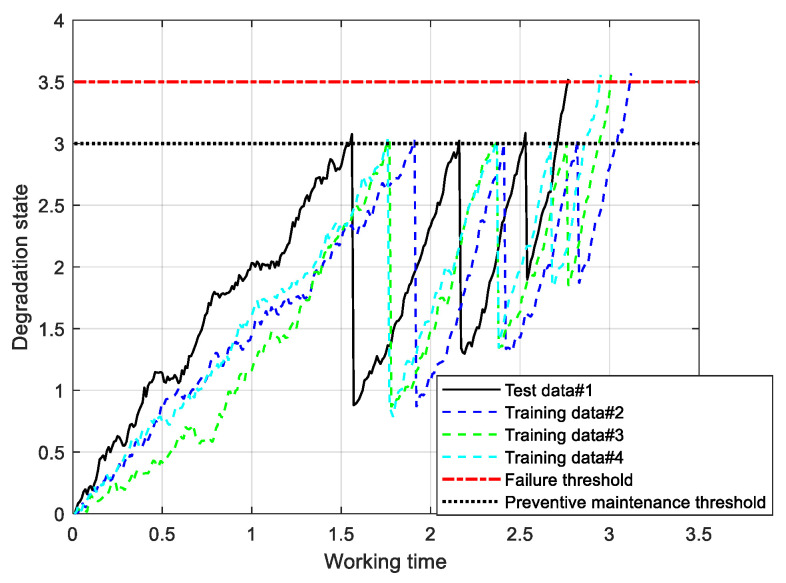
The degradation trajectory from numerical simulation.

The degradation trajectory for the model described in Equation (6) can be generated through simulation using the Euler discretization method [33], while the model parameters are specified in Table 1. Firstly, let $\mu \left(\tau ,\theta \right)$ denote the power-law function $t^{\theta }$. By incorporating the model parameters from Table 1 into the degradation model, we can obtain four degradation trajectories, as depicted in Figure 2. The black solid line, #1, indicates the online test data, which are employed to verify the effect of the proposed method. The other dotted lines, #2–4, represent the historical degradation data that are employed for the offline estimation of model parameters.

**Figure 3:**
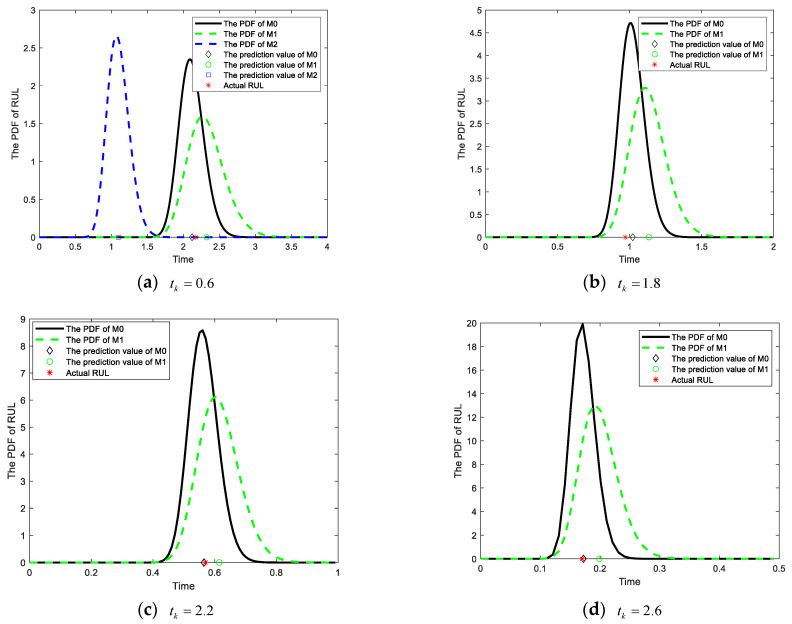
The PDFs and prediction results of RUL at the various time points.

Based on the degradation data from the numerical simulation, the results of RUL prediction can be obtained by M0, M1, and M2. In this study, four monitoring points ($t_{k}=0.6$, $t_{k}=1.8$, $t_{k}=2.2$, and $t_{k}=2.6$) were carefully selected for RUL prediction in the four degradation stages. In order to compare the accuracy of prediction results, Figure 3 provides the PDFs of RUL obtained from different methods. The solid black line represents the PDF of RUL obtained from M0, while the dotted green line corresponds to the PDF of RUL for M1, which is an imperfect maintenance method discussed in [25,27]. Additionally, the dotted blue line illustrates the PDF of RUL for M2, which does not account for any influence from maintenance activities.

**Figure 4:**
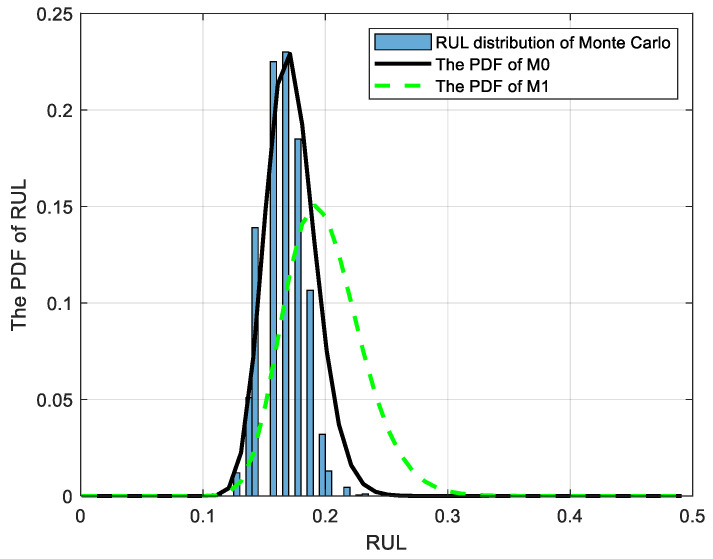
The comparison of RUL distributions.

When the monitoring time $t_{k}=0.6$, the device has not undergone any maintenance activities, and the RUL at the corresponding time can be obtained from M0, M1, and M2. It can be observed from Figure 3a that M2 has disregarded the impact of subsequent maintenance activities on the degradation process. From the initial stage until reaching the failure threshold ω, there is a significant disparity between the predicted RUL and the actual RUL. The analysis of Figure 3 reveals that, in comparison with method M1, the proposed method M0 not only incorporates the influence of maintenance activities on the degradation process but also accounts for the variability in adaptive drift terms in future degradation processes. Consequently, the PDF of M0 exhibits a narrower and more pronounced shape, indicating reduced uncertainty in prediction results and closer alignment between the predicted RUL and the actual RUL. With the accumulation of degradation data on test samples, M0 can ensure the model parameters are continuously updated so that the RUL results always maintain a high prediction accuracy. In addition, to demonstrate the superiority of the adaptive Wiener process in the proposed method M0 over the AR(1) model in M1, the RUL distribution was obtained through Monte Carlo simulations of 2000 degenerate trajectories. The resulting distributions were then compared with the PDFs of methods M0 and M1, as illustrated in Figure 4. It is evident from the figure that the PDF of RUL obtained using method M0 aligns more closely with the RUL distribution derived from the Monte Carlo method. This suggests that the adaptive Wiener process model employed in M0 is more effective than the AR(1) model used in M1 for degradation modeling.

To quantitatively analyze the RUL prediction results of these three methods, this paper presents the relative error (RE) and mean squared error (MSE) [34] of different methods at four monitoring points, as shown in Table 2. It can be observed from Table 2 that the proposed method M0 consistently exhibits superior prediction accuracy for RUL compared to method M1, with the former closely approximating the actual RUL. As a crucial metric for quantifying the uncertainty of prediction results, the smaller MSE corresponds to better-predicted results. The results presented in Table 2 demonstrate that the MSE of the proposed method M0 is consistently smaller than those of methods M1 and M2, thereby indicating superior prediction uncertainty.

**Figure 5:**
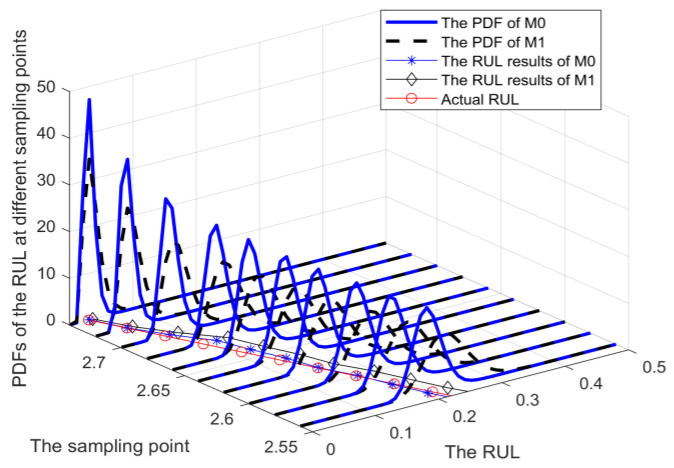
The PDFs and prediction results of RUL.

After conducting three preventive maintenance activities, M0 and M1 were employed to predict the RUL of the device during the fourth stage, with detailed outcomes presented in Figure 5. It is not difficult to find from Figure 4 that, compared with the RUL prediction result obtained from M1, the RUL expectation of the proposed method M0 is closer to the actual RUL, and M0 demonstrates reduced uncertainty in the PDF of RUL. Through the aforementioned comparison, it is evident that, in contrast to method M1, the proposed method M0 incorporates considerations for the adaptive drift variability of the model, resulting in enhanced precision and reduced uncertainty in predicting RUL.

### 5.2. A Case Study of the Gyroscope

The performance of a gyroscope, which is the core component of an inertial navigation system (INS), directly affects the guidance accuracy and even the overall performance of the control system. During the operation of an INS, the high-speed rotation of the gyroscope rotor inevitably induces motor shaft wear. With the accumulation of working time, the drift coefficient of the gyroscope will increase from the deformation of the motor bearing. When the drift coefficient exceeds the threshold, it indicates the gyroscope’s degradation and failure.

In order to prolong the operational duration of the gyroscope and ensure the secure and stable functioning of the INS, preventive maintenance activities are typically scheduled as follows. When the gyro drift coefficient exceeds the predetermined threshold for preventive maintenance, the hardware compensation circuit is utilized to effectively mitigate both the constant and primary terms of gyro drift. Considering that the majority of maintenance activities in practical engineering are characterized by imperfections, it is expected that the residual degradation state and degradation rate of devices will progressively increase following maintenance activities. When the maintenance effect fails to meet the operational requirements, the gyroscope will be replaced with spare parts. However, there is a limited number of maintenance activities throughout the entire degradation process.

**Figure 6:**
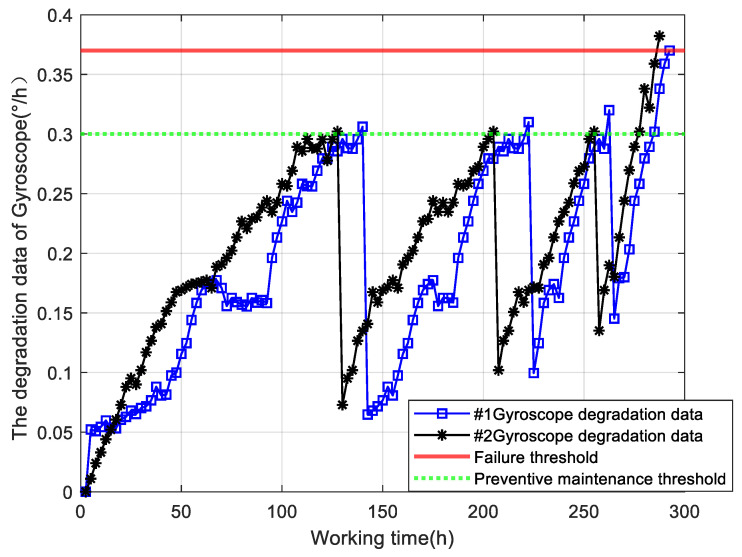
The degradation data under maintenance activities.

The proposed method is verified in a practical case using the degradation data of an electromechanical gyroscope, as depicted in Figure 6. The degradation monitoring data of two gyroscopes are provided, which were monitored at a time interval of 2.5 h. According to the technical specifications of this type of inertial navigation system, the gyroscope drift coefficient failure threshold should be 0.37 (°)/h, while the preventive maintenance threshold should be set at 0.3 (°)/h. The degradation process, as depicted in Figure 6, underwent three rounds of correction and maintenance. Specifically, gyroscope #1 was monitored at 117 points, with an actual operational life of 282.5 h, while gyroscope #2 was monitored at 115 points, with an actual operational life of 277.5 h. The degradation data of the gyroscope gradually increase over time, and proactive maintenance activities should be conducted on the gyroscope when it reaches the preventive maintenance threshold to enhance its degradation state through compensation correction and other measures. However, residual degradation still persists, and the rate of degradation escalates further in subsequent stages. Further maintenance activities will not be conducted until the failure threshold is reached after completing the predetermined number of proactive maintenance tasks.

**Figure 7:**
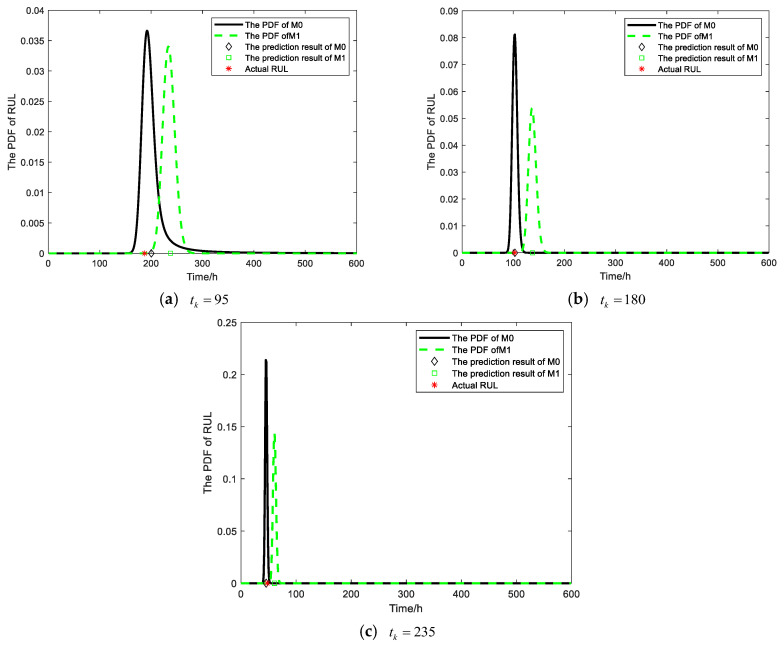
The PDFs and prediction results of RUL for gyroscope #1.

The degradation data of gyroscope #1 were employed as test samples in this paper to validate the proposed method M0. The RUL prediction results of the M0 and M1 methods can be compared after obtaining the model parameters $\Theta =\left\{a,b,\lambda_{10},\mu_{\zeta ,}\sigma_{\zeta }^{2},\kappa ,\sigma_{B}^{2}\right\}$ through the parameter estimation method outlined in Section 4. Firstly, a monitoring point is selected in each of the first three stages to predict the RUL of gyroscope #1. The PDFs and prediction results of RUL are depicted in Figure 7.

**Figure 8:**
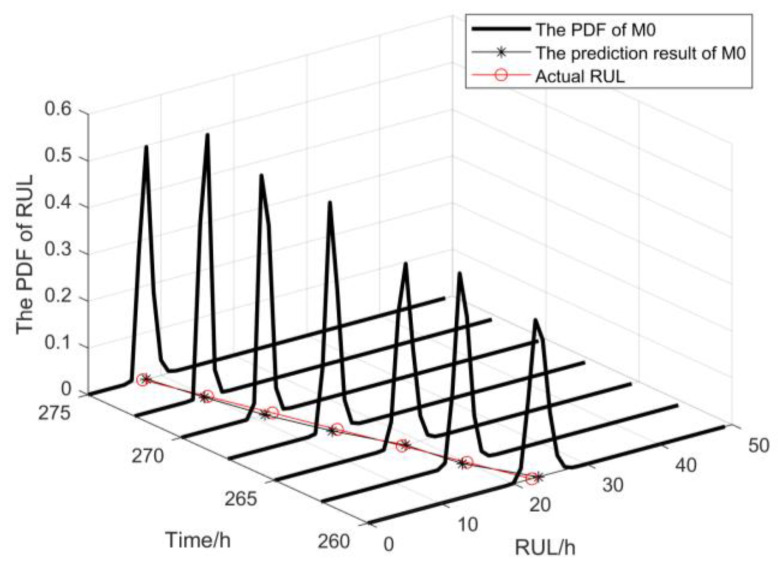
The PDF of RUL in the last stage.

The PDF of RUL for the proposed method M0 exhibits a narrower and sharper distribution compared to method M1, as depicted in Figure 7, across various monitoring points during the initial three stages. Moreover, the prediction results of M0 demonstrate a closer proximity to the actual RUL. These findings collectively indicate that method M0 offers higher prediction accuracy and reduced uncertainty in RUL prediction. After three rounds of maintenance activities, two prediction methods were employed to predict the RUL of the gyroscope during its final degradation stage. The PDF generated by the proposed method M0 for predicting RUL at various monitoring points is illustrated in Figure 8.

**Figure 9:**
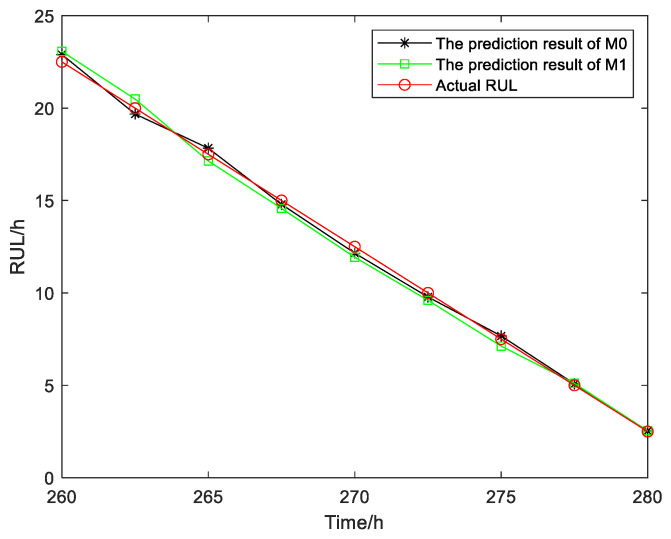
The RUL prediction results of gyroscope # 1.

As shown in Figure 8, the proposed method M0 takes into account the variability in the adaptive drift term in the future, and its RUL prediction is close to the actual RUL of the device. The specific RUL prediction results, MSE, and the prediction Score corresponding to the two methods at different monitoring points are provided in this paper for qualitative and quantitative comparison of their prediction performance. The comparison between the two methods is illustrated in Figure 9 and Table 3. In practical engineering applications, underestimating the predicted RUL value may result in a relatively conservative maintenance strategy, while overestimating the predicted RUL value may lead to catastrophic accidents caused by sudden device failure. The MSE and Score are two crucial metrics for evaluating the accuracy and distribution uncertainty of RUL prediction outcomes. A smaller MSE value indicates a more concentrated distribution of RUL predictions around the actual values, implying reduced uncertainty in the predicted RUL. It can be clearly observed from Table 3 that compared with the prediction result of the M1 method, the proposed method M0 has a smaller Score value, and the deviation and MSE of the prediction result of the M0 method are significantly smaller than those of the M1 method at several monitoring points. Therefore, the proposed method M0 exhibits higher accuracy in predicting the RUL and lower uncertainty in its predictions.

## 6. Conclusions

The RUL prediction problem for repairable degrading devices considering the impact of imperfect maintenance activities has been investigated in this paper. An adaptive degradation model incorporating the impact of imperfect maintenance was established by utilizing Brownian motion for adaptive drift. It not only integrated maintenance activities into degradation modeling but also facilitated adaptive dynamic prediction during future processes. Subsequently, the RUL expression of the proposed model was derived based on the concept of FHT, which achieved real-time RUL prediction under imperfect maintenance. Furthermore, an algorithm for parameter identification was introduced that employs MLE for offline estimation, while a Bayesian posterior estimation was developed for online updating. Finally, the effectiveness of the proposed method was verified through cases of numerical simulation and gyroscopes. Furthermore, several directions warrant further exploration in future research. Firstly, it is imperative to investigate the impact of condition monitoring to facilitate wider application scenarios. Secondly, researching optimal health management strategies is crucial, including joint decisions for preventive maintenance and spare parts inventory.

## Figures and Tables

**Table 1:** The values of model parameters.

Parameter	Values	Parameter	Values
Initial drift term $\lambda_{10}$	1.8	Residual degradation hyperparameter α	2
Diffusion coefficient of drift term κ	0.1	Residual degradation hyperparameter β	5
Change coefficient means $\mu_{\zeta }$	1.2	Maintenance frequency N	3
Standard deviation of coefficient change $\sigma_{\zeta }$	0.3	Failure threshold ω	3.5
Diffusion coefficient $\sigma_{B}$	0.35	Preventive maintenance threshold $\omega_{p}$	3
Time interval Δt	0.01	Nonlinear parameter θ	1.2

**Table 2:** Relative error and mean squared error of RUL prediction.

Time $t_{k}$	RE of RUL Prediction			MSE of RUL Prediction		
	M0	M1	M2	M0	M1	M2
0.6	2.30%	11.62%	44.74%	0.0025	0.0626	0.9424
1.8	5.31%	16.87%	\	0.0026	0.0268	\
2.2	0.85%	7.82%	\	2.34 × 10^−5^	0.002	\
2.6	1.27%	17.05%	\	4.67 × 10^−6^	8.40 × 10^−4^	\

**Table 3:** The MSE and Score of the RUL prediction.

Monitoring Point/h	M0	M1	Actual RUL/h
	MSE	MSE	
260	0.1600	0.3260	22.5
262.5	0.1024	0.2401	20
265	0.1089	0.1296	17.5
267.5	0.0441	0.1849	15
270	0.1225	0.3249	12.5
272.5	0.0484	0.1600	10
275	0.0289	0.1444	7.5
277.5	0.0062	0.0115	5
280	0.00089	0.0014	2.5
Score	5.2228	5.6447	\

## Data Availability

The data that support the findings of this study are available from the corresponding author upon reasonable request.

## Acronyms, Abbreviations and Nomenclatures

**Acronyms and Abbreviations**	
PdM	predictive maintenance
RUL	remaining useful life
PDF	probability density function
CDF	cumulative density function
AR(1)	autoregressive model of order 1
FHT	first hitting time
CM	condition monitoring
MLE	maximum likelihood estimation
RE	relative error
MSE	mean squared error
INS	inertial navigation system
**Nomenclatures**	
ω	failure threshold
$\omega_{p}$	preventive maintenance threshold
$X\left(t\right)$	degradation state
$\lambda \left(t\right)$	drift term
$\sigma_{B}$	diffusion coefficient
$B\left(t\right)$	standard Brownian motion
$z_{i}$	residual degradation state
$\zeta_{i}$	change coefficient
